# Relation between regional and global systolic function in patients with ischemic cardiomyopathy after β-Blocker therapy or revascularization

**DOI:** 10.1186/1532-429X-12-7

**Published:** 2010-01-27

**Authors:** TAM Kaandorp, JJ Bax, SE Bleeker, J Doornbos, EP Viergever, D Poldermans, EE van der Wall, A de Roos, HJ Lamb

**Affiliations:** 1 Department of Radiology, Leiden University Medical Center, the Netherlands; 2 Department of Cardiology, Leiden University Medical Center, the Netherlands; 3 Regional Public Health Laboratory Kennemerland, Haarlem, the Netherlands; 4 ThoraxCenter Rotterdam, the Netherlands

## Abstract

**Background:**

To assess the relationship between improved regional and global myocardial function in patients with ischemic cardiomyopathy in response to β-blocker therapy or revascularization.

**Materials and methods:**

Cardiovascular Magnetic Resonance (CMR) was performed in 32 patients with ischemic cardiomyopathy before and 8 ± 2 months after therapy. Patients were assigned clinically to β-blocker therapy (n = 20) or revascularization (n = 12). CMR at baseline was performed to assess regional and global LV function at rest and under low-dose dobutamine. Wall thickening was analyzed in dysfunctional, adjacent, and remote segments. Follow-up CMR included rest function evaluation.

**Results:**

Augmentation of wall thickening during dobutamine at baseline was similar in dysfunctional, adjacent and remote segments in both patient groups. Therefore, baseline characteristics were similar for both patient groups. In both patient groups resting LV ejection fraction and end-systolic volume improved significantly (p < 0.05) at follow-up. Stepwise multivariate analysis revealed that improvement in global LV ejection fraction in the β-blocker treated patients was significantly related to improved function of remote myocardium (p < 0.05), whereas in the revascularized patients improved function in dysfunctional and adjacent segments was more pronounced (p < 0.05).

**Conclusion:**

In patients with chronic ischemic LV dysfunction, β-Blocker therapy or revascularization resulted in a similar improvement of global systolic LV function. However, after β-blocker therapy, improved global systolic function was mainly related to improved contraction of remote myocardium, whereas after revascularization the dysfunctional and adjacent regions contributed predominantly to the improved global systolic function.

## Introduction

Recent estimations reveal that 4.9 million patients suffer from chronic heart failure in the United States, indicating the magnitude of this major health care problem [1]. Ischemic cardiomyopathy is a frequent cause of chronic heart failure. Different treatment options are available for the treatment of ischemic cardiomyopathy, including medical therapy and revascularization.

Beta-blocking agents have shown substantial benefit in patients with various degrees of heart failure [2-4]. The mechanisms by which β-blockers reduce mortality among heart failure patients remain unclear. Heart failure is a complex disease that is characterized by chronic excessive sympathetic nervous system stimulation causing myocardial toxicity and further depression of left ventricular (LV) function [5]. It is suggested that LV function improves after β-blocker therapy as a result of reversal of catecholamine-mediated myocardial toxicity in the partially viable or noninfarcted regions of the LV and possibly by improving function in regions of hibernating myocardium [6]. It has been suggested that dobutamine induced improvement in segmental contraction of dysfunctional myocardium before treatment is related to improved global LV function after medical therapy [7,8]. However, remote myocardium may potentially also contribute to the improvement of LV function after therapy, but this contribution has not yet been evaluated.

The beneficial effect of revascularization of dysfunctional myocardium in patients with ischemic cardiomyopathy has traditionally been measured by its effect on improvement of resting regional and global LV function [9,10]. Revascularization is expected to improve regional function when viable, but jeopardized myocardium is present in an area of dysfunctional myocardium [11]. Furthermore, it has been recognized that LV end-systolic volume predicts long-term outcome to best advantage after revascularization [12].

Cardiovascular magnetic resonance (CMR) is a validated and reliable method to assess global and regional myocardial function in normal and diseased hearts. Functional CMR is well suited to assess resting wall motion [13-17] as well as the response to dobutamine for predicting viability of dysfunctional myocardial segments [18].

We sought to define the contribution of regional myocardial segments to the improvement of global systolic LV function in patients after medical therapy or revascularization. We hypothesized that a differential effect on regional myocardial segments occurs depending on the type of therapy. Systemic medical therapy is expected to have a more global effect on both ischemic, dysfunctional myocardial segments and on non-ischemic, remote myocardium, whereas revascularization will have a more local effect on the ischemic, dysfunctional myocardium depending on the territory of the revascularized vessels.

Therefore, the purpose of the present study was to assess the relationship between improved regional and global myocardial function in patients with ischemic cardiomyopathy in response to β-blocker therapy or revascularization.

## Materials and methods

### Patient population

Thirty-two patients with chronic ischemic cardiomyopathy and LV ejection fraction (EF) <40% on gated resting Tc-99m-SPECT, were included. All patients were in sinus rhythm. Patients with a recent infarction (<3 months), unstable angina, valvular disease pacemakers and/or intracranial clips were excluded.

Patients were included consecutively. Patients that did not qualify for revascularization were assigned to the β-blocker therapy group. Patients did not qualify for revascularization because: 1. Patients had poor target vessels (small vessels, not amendable for revascularization) 2. Patients had prior existing co-morbidity (e.g. renal failure); 3. Patients refused to undergo revascularization. β-blocker therapy was started at an initial dose of 3.125 mg carvedilol twice daily. Subsequently, carvedilol was titrated at 1-week intervals as tolerated, up to target dose of 25 mg twice daily [19].

β-Blocker treated patients were compared to patients who underwent revascularization. In the revascularization patients, coronary artery bypass surgery was performed in 75% and percutaneous coronary intervention in 25%. Each patient gave informed consent to the study protocol that was approved by the local ethics committee.

### CMR acquisition

Patients were studied by CMR before therapy and at 8 ± 2 months after therapy. At baseline, the CMR protocol consisted of a resting cine CMR, Late Gadolinium Enhancement (LGE) and low-dose dobutamine (10 μg/kg/min) cine CMR. Follow-up CMR included rest function evaluation only. The congestive heart failure classification by the New York Heart Association (NYHA) was determined at baseline and follow-up by the patient's cardiologist, who was blinded to the CMR data.

A 1.5-Tesla Gyroscan ACS-NT CMR scanner (Philips Medical Systems, The Netherlands) and a 5 elements cardiac synergy coil were used. Patients were studied in supine position. All images were acquired during breath-holds of approximately 15 seconds and were gated to the vector ECG; blood pressure was monitored continuously during the examination (Millennia, Invivo Research, Orlando Fla, USA). The heart was imaged from apex to base with 10 to 12 imaging levels (dependent on heart size) in short-axis view using a steady-state free-precession sequence. Typical acquisition parameters were: field of view 400 × 400 mm^2^, matrix size 256 × 256, slice thickness 10.00 mm, flip angle 50°, time to echo 1.82 ms and time to repeat 3.65 ms. Temporal resolution was 25-39 ms, depending on heart rate. Geometry settings of rest cine CMR scans were stored and repeated for LGE and low-dose dobutamine stress CMR.

LGE images were acquired approximately 15 minutes after bolus injection of gadopentate dimeglumine (Gadolinium-DTPA, Magnevist, Schering/Berlin, Germany, 0.15 mmol/kg) with an inversion-recovery gradient echo sequence; inversion time was determined using real time planscan. Typical parameters were the following: field of view 400 × 400 mm^2^, matrix size 256 × 256, slice thickness 5.00 mm, slice gap - 5 mm, flip angle 15°, time to echo 1.36 ms and time to repeat 4.53 ms.

For evaluation of myocardial function under pharmacological stress, intravenous dobutamine infusion was started at a rate of 5 μg/kg/min and increased after 5 min to 10 μg/kg/min. Then, after 5 min (at steady state), low-dose dobutamine images were acquired in 2- and 4-chamber and short-axis views. The same parameters were applied as described for rest imaging.

### CMR image analysis

Data were analyzed on a remote workstation using MASS software (MASS, Medis, The Netherlands). The endo- and epicardial borders of the end-diastolic and end-systolic frames were manually traced with exclusion of papillary muscles, trabeculae, and epicardial fat [20]. LV end-diastolic and end-systolic volumes were calculated and LVEF was derived.

The amount of infarcted tissue was determined by drawing (manually) regions of interest around the scar tissue. In addition, the percentage of myocardium was calculated that was affected by infarction, relative to the total LV mass. LGE images were scored visually by two experienced observers (blinded to other CMR and clinical data) using a 17-segment model. Each segment was graded on a 5-point scale (segmental scar score) with 0: absence of LGE, 1: LGE of 1-25% of LV wall thickness, 2: LGE extending to 26-50%, 3: LGE extending to 51-75%, and 4: LGE extending to 76-100% of the LV wall thickness [21].

To determine regional wall motion at rest, cine CMR images were visually interpreted by two experienced observers (blinded to other CMR and clinical data) using a 17-segment model. Each segment was assigned a wall motion score using a 5-point scale with 0: normal wall motion, 1: mild hypokinesia, 2: severe hypokinesia, 3: akinesia, and 4: dyskinesia [22]. In the dysfunctional segments at rest (score 1-4), the presence or absence of contractile reserve was based on visual analysis of the difference in myocardial wall motion between CMR acquisitions at rest and during infusion of low-dose dobutamine. An improvement in segmental wall motion score by 1 grade or more was considered indicative of contractile reserve.

**Figure 1:**
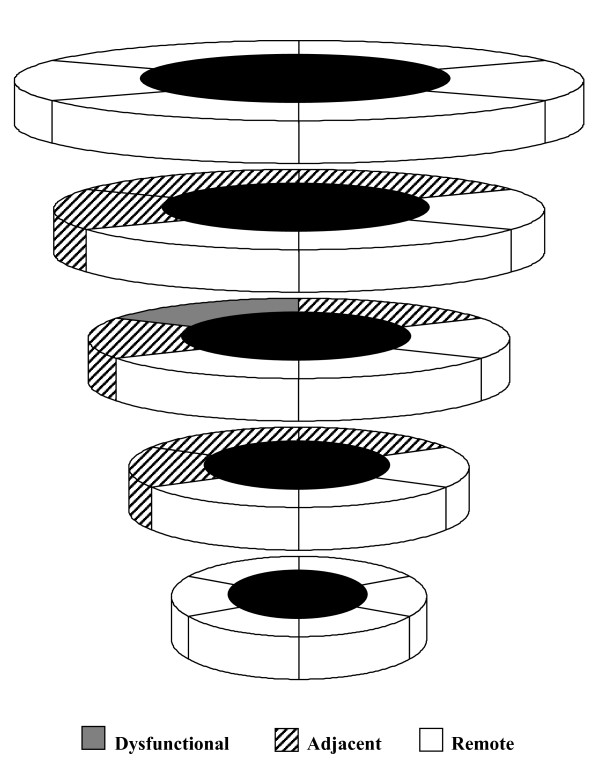
**Diagram in 3D showing the relative position in a virtual left ventricle of the three regions that were quantified using the centreline method**. Definition of segments was based on a visual wall motion score using a 17-segment model and a 5-point scoring system with 0: normal wall motion, 1: mild hypokinesia, 2: severe hypokinesia, 3: akinesia, and 4: dyskinesia. Myocardial segments with a visual wall motion score from 1 to 4 were considered as dysfunctional segments (grey). The myocardial segments next to these dysfunctional segments in 3-dimensions, were considered as adjacent segments (dashed). The remaining myocardial segments were considered as remote tissue (white).

Myocardial segments with a visual wall motion score at rest from 1 to 4 were considered as dysfunctional segments. The myocardial segments next to these dysfunctional segments in 3-dimensions, were considered as adjacent segments. The remaining myocardial segments were considered as remote tissue (Figure 1). Wall thickness was then quantified in the above defined three regions: (1) dysfunctional, (2) adjacent, and (3) remote segments, using the centerline method as described before [23]. Wall thickening was calculated based on the difference in wall thickness between end-diastole and end-systole. The change in wall thickening was calculated between baseline and dobutamine stress acquisitions, or between baseline and follow-up acquisitions.

### Statistical analysis

Continuous data were expressed as mean ± SD and compared using the Student's t test for (un-) paired data when appropriate. Stepwise multivariate analysis was performed to determine the relation between regional improvement in myocardial function and improvement in global LVEF at follow up. All tests were two-tailed and a P-value < 0.05 was considered statistically significant.

The authors had full access to the data and take responsibility for its integrity. All authors have read and agree to the manuscript as written.

## Results

### Baseline

Baseline characteristics are summarized in Table 1. The variables listed in Table 1 were not statistically significantly different between patients in the β-blocker and revascularization groups (Table 1). Table 2 summarizes LV dimensions and global systolic function. At baseline, there were no statistically significant differences between both patient groups (Table 2). Table 3 shows scar burden and scar morphology. At baseline, there were no statistically significant differences between both patient groups (Table 3).

**Table 1:** Baseline characteristics of the study population

	β-Blocker therapy	Revascularization
	(n = 20)	(n = 12)
Age (yrs)	67 ± 8	68 ± 6
Gender (male/female)	20/0	11/1
Time to follow-up (months)	8 ± 3	9 ± 4
Nr of stenosed (> 70%) coronary arteries	2.7 ± 0.5	2.5 ± 0.5
Nr of segments with contractile reserve	5.5 ± 3.2	3.7 ± 1.7
NYHA classification	2.3 ± 0.5	2.2 ± 0.5

*Medication:*		
Asperin	95%	100%
Angiotensin converting enzyme inhibitors	65%	58%
Diuretics	55%	50%
Nitrates	35%	50%
*Nr of segments with:*		
Dysfunctional tissue	7.4 ± 3.2	5.5 ± 1.5
Adjacent tissue	6.3 ± 2.0	6.5 ± 1.1
Remote tissue	3.4 ± 2.6	5.1 ± 2.0

All comparisons between both groups were statistically non-significant.

**Table 2:** Effect of therapy on left ventricular dimensions and global systolic function

	β-blocker therapy		Revascularization	
	Baseline	Follow-up	Baseline	Follow-up
LVEDV (ml)	271 ± 63	254 ± 55	238 ± 48	250 ± 59
LVESV (ml)	190 ± 63	163 ± 54*	152 ± 35	140 ± 41*
LVEF (%)	31 ± 7	37 ± 9*	36 ± 6	44 ± 6*

LVEDV: left ventricular end-diastolic volume; LVESV: left ventricular end-diastolic volume; LVEF: left ventricular ejection fraction.

*: P < 0.05 for baseline versus follow-up values. Other comparisons were non-significant.

**Table 3:** Scar morphology at baseline.

	β-blocker therapy	Revascularization
Scar tissue on LGE CMR (g)	31.9 ± 18	29.5 ± 15.0
Scar tissue on LGE CMR (%)	19.6 ± 10.9	17.1 ± 7.5

*Nr of segments with:*		

LGE score 0	8.8 ± 3.9	10.6 ± 1.9
LGE score 1	3.9 ± 3.1	2.8 ± 2.4
LGE score 2	1.9 ± 1.6	1.2 ± 1.1
LGE score 3	1.5 ± 1.6	1.7 ± 1.8
LGE score 4	1.0 ± 1.4	0.8 ± 1.2

LGE Score: 0: absence of LGE, 1: LGE of 1-25% of LV wall thickness, 2: LGE extending to 26-50%, 3: LGE extending to 51-75%, and 4: LGE extending to 76-100% of the LV wall thickness. There were no statistically significant differences between both patient groups.

**Figure 2:**
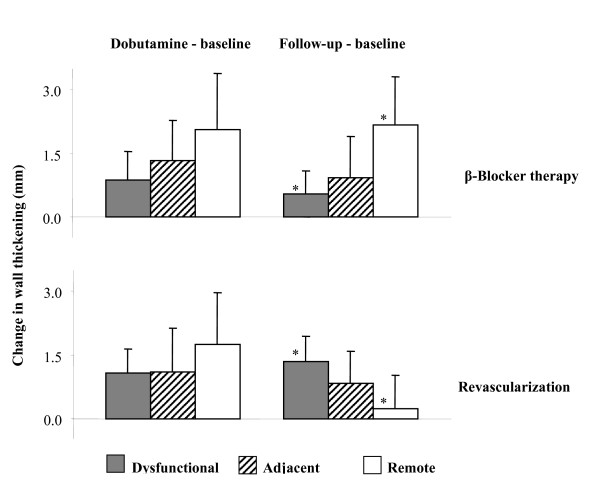
**Changes in wall thickening from baseline to low-dose dobutamine (left panel) or from baseline to follow-up (right panel) for patients treated with β-blockers (upper panel) or undergoing revascularization (lower panel)**. Note the similarity in regional distribution when comparing dobutamine-baseline measurements of both patient groups. In addition, note that the regional distribution is similar in response to dobutamine or β-blocker therapy. Moreover, in patients treated with β-blockers, the remote tissue contributed predominantly to overall wall thickening, whereas after revascularization the dysfunctional segments contributed most to the observed changes in systolic wall thickening. *: P ≤ 0.05 for β-blocker therapy and revascularization.

The response to low-dose dobutamine at baseline in the dysfunctional, adjacent, and remote myocardial segments is summarized in Figure 2 (left panel). There were no statistically significant differences in wall thickening in any myocardial segment, when comparing the β-blocker and revascularization groups at baseline (Figure 2).

### Follow-up after β-blocker or revascularization therapy

Clinical assessment revealed that the NYHA classification changed from 2.3 ± 0.5 at baseline to 1.8 ± 0.6 at follow-up (P < 0.05) in the β-blocker treated patients, and from 2.2 ± 0.5 to 1.5 ± 0.5 in the revascularization patient group (P < 0.05). After β-blocker or revascularization therapy, LV end-diastolic volume did not change significantly in both patient groups while LV end-systolic volume and LVEF improved significantly in both the β-blocker and revascularization groups to the same extent (Table 2).

Comparison of myocardial wall thickening between follow-up and baseline, revealed that in the β-blocker group the remote segments showed the largest improvement (Figure 2, right upper panel). Stepwise multivariate analysis in β-blocker patients revealed that improvement in LVEF after therapy was mainly related to improvement in function of the remote region (y = 2 × Rmt + 1.8, where Rmt = difference in wall thickening at rest in remote tissue between follow-up and baseline, P= 0.002, acceptance value 0.05). In the revascularization patient group, most improvement in myocardial wall thickening between follow-up and baseline was achieved in the dysfunctional segments (Figure 2, right lower panel). Stepwise multivariate analysis in revascularization patients revealed that improvement in LVEF was mainly related to improvement in function of the dysfunctional and adjacent segments (y = 2.6 × Dsf + 1.8 × Adj + 0.6, where Dsf = difference in wall thickening at rest in dysfunctional segments between follow-up and baseline, Adj = difference in wall thickening at rest in adjacent segments between follow-up and baseline, P= 0.001, acceptance value 0.05).

**Figure 3:**
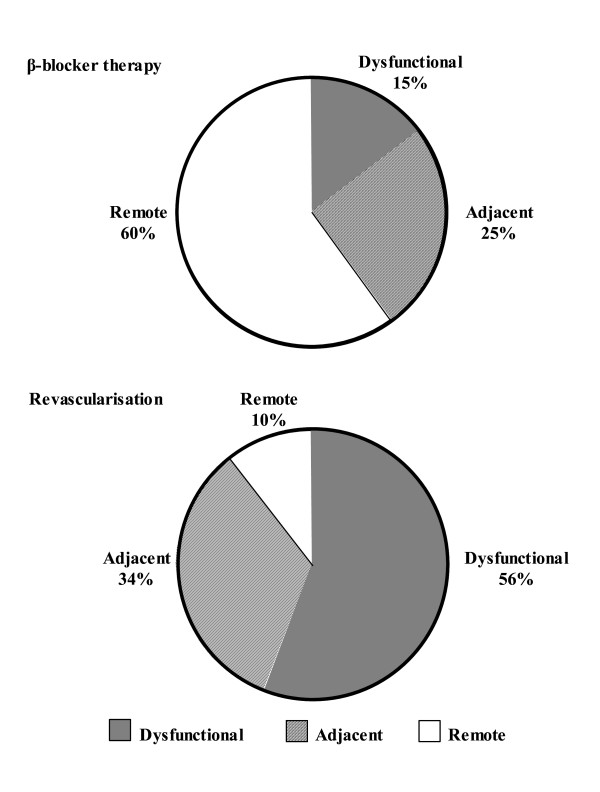
**Relative contributions of dysfunctional, adjacent and remote tissue to overall systolic wall thickening changes after therapy**. Note the inverse regional contributions: remote tissue contributed most to the effect of β-blocker therapy, whereas dysfunctional segments contributed mainly to the effect of revascularization.

Direct comparison between the β-blocker and revascularization groups concerning the myocardial segments shows a statistically significant difference (P < 0.05) in wall thickening between the dysfunctional and remote segments (Figure 2 right panel, top and bottom). As a consequence, a reversed pattern is observed in the contribution of the dysfunctional and remote myocardial segments to improvement in wall thickening after β-blocker or revascularization therapy. This differential pattern is also illustrated in Figure 3, showing the relative contributions of the myocardial segments to improvement in LVEF after β-blocker or revascularization therapy. In the β-blocker treated patients (Figure 3, top), the remote tissue contributes for 60% to the improvement in LVEF, whereas in the revascularized patients (Figure 3, bottom), the dysfunctional segments contributed for 56% to the improvement in LVEF after therapy.

## Discussion

The present study demonstrates the relation between regional and global myocardial function in patients with ischemic cardiomyopathy after β-blocker or revascularization therapy. Following β-blocker therapy, improved global systolic function is mainly related to improved contraction of remote myocardium, whereas after revascularization the dysfunctional and adjacent regions contributed most to the improvement in global systolic function.

### Baseline

At baseline, NYHA classification was similar for β-blocker treated and revascularization patients. In addition, global systolic function, the % scar tissue or the number of segments with contractile reserve were similar in both patient groups at baseline. Furthermore, both patient groups showed the same response to low-dose dobutamine at baseline in the dysfunctional, adjacent, and remote myocardial segments. Accordingly, the clinically defined patient groups were comparable at baseline concerning myocardial function.

### Follow up after β-blocker or revascularization therapy

Following β-blocker or revascularization therapy, LV systolic function improved significantly, and to a similar extent. Accordingly, the global functional response to therapy was comparable between both patient groups. This finding is consistent with results from previous studies [5,6,9,12,24,25]. Furthermore, improvement in LVEF was accompanied by an improvement in NYHA classification from baseline to follow up, to a similar extent in both patient groups.

Stepwise multivariate analysis in β-blocker patients revealed that improvement in LVEF after therapy was mainly related to improvement in function of the remote myocardium. The present results concerning β-blocker therapy effect on remote tissue are in agreement with previous studies by Reiken et al. Both in a canine model [26] and in a patient study (performed in explanted hearts) [27], the authors showed that β-blockers normalized Ca^++^-channel function in failing myocardium. Remote myocardium in the current study may be regarded as myocardium with relatively preserved myocyte function as compared to adjacent and dysfunctional regions.

However, in remote tissue, myocardial hypertrophy may develop when excessive pressure or volume overload is imposed to sustain the burden of a dysfunctional segment [28]. The remote hypertrophied tissue may appear to contract normally, but could in fact represent "pseudonormalized" myocardium. Therefore, the relatively mildly failing remote myocardium can still show a positive response to restoration of the Ca^++^-channel function by administration of β-blockers, whereas the adjacent and dysfunctional tissue cannot. Furthermore, recent data demonstrated that the effect of β-blocker therapy could be predicted by the increase in LVEF during low-dose dobutamine infusion [29]. Since dobutamine is a β-receptor agonist, it may be able to temporarily mimic sympatic nervous system stimulation, and predict the effect of β-blockers on LVEF. Interestingly, the regional response pattern after β-blocker therapy in the current study (Figure 2, right upper panel) is similar to the regional response to dobutamine in β-blocker treated patients (left upper panel). The latter observation supports the hypothesis that low-dose dobutamine infusion in patients with chronic ischemic LV dysfunction may predict clinical outcome after β-blocker therapy [29].

Stepwise multivariate analysis in revascularization patients revealed that improvement in LVEF was mainly related to improvement in function of the dysfunctional and adjacent myocardial segments. This observation is in agreement with data obtained in previous studies [30,31]. Recovery of hibernating myocardium occurs after successful revascularization [11,32,33]. In the present study, no improvement in wall thickening was noted in the remote region in the revascularization patients. The latter finding may be explained by the fact that only blood flow to dysfunctional and surrounding adjacent tissue is restored. As a result, no short-term improvement in function can be expected in the remote area. An interesting finding in the present study was that adjacent myocardium also contributed significantly to the improvement of global systolic function after revascularization therapy.

### Linitations

The number of patients was relatively small and therefore the observations need confirmation in larger cohorts. Myocardial tagging was not performed; this could have provided a more detailed strain assessment across the myocardial segments of interest.

## Conclusion

In patients with chronic ischemic LV dysfunction, β-Blocker therapy or revascularization resulted in a similar improvement of global systolic LV function. However, after β-blocker therapy, improved global systolic function was mainly related to improved contraction of remote myocardium, whereas after revascularization the dysfunctional and adjacent regions contributed predominantly to the improved global systolic function.

## Competing interests

The authors declare that they have no competing interests.

## Authors' contributions

TK wrote the manuscript, designed and carried out the study; JB, HL have been involved in writing the article and made substantial contributions to it's conception and design. SB participated in the design of the study and performed the statistical analysis; DP, EV, EvdW and JD have made substantial contributions to acquisition and interpretation of data; AdR conceived of the study, and participated in its design and coordination and helped to draft the manuscript. All authors read and approved the final manuscript.
